# Pancreatic stone protein point-of-care testing can reduce healthcare expenditure in sepsis

**DOI:** 10.1186/s13561-022-00381-z

**Published:** 2022-07-22

**Authors:** John E. Schneider, Katherine Dick, Jacie T. Cooper, Nadine Chami

**Affiliations:** 1 Avalon Health Economics, 119 Washington Street, Morristown, NJ 07960 USA; 2 Ontario Medical Association, Toronto, Ontario Canada

**Keywords:** Sepsis, Pancreatic stone protein, Point-of-care testing, Antibiotic stewardship, Cost impact

## Abstract

**Background:**

Sepsis is a life-threatening organ dysfunction in response to infection. Early recognition and rapid treatment are critical to patient outcomes and cost savings, but sepsis is difficult to diagnose because of its non-specific symptoms. Biomarkers such as pancreatic stone protein (PSP) offer rapid results with greater sensitivity and specificity than standard laboratory tests.

**Methods:**

This study developed a decision tree model to compare a rapid PSP test to standard of care in the emergency department (ED) and intensive care unit (ICU) to diagnose patients with suspected sepsis. Key model parameters included length of hospital and ICU stay, readmission due to infection, cost of sepsis testing, length of antibiotic treatment, antibiotic resistance, and clostridium difficile infections. Model inputs were determined by review of sepsis literature.

**Results:**

The rapid PSP test was found to reduce costs by $1688 per patient in the ED and $3315 per patient in the ICU compared to standard of care. Cost reductions were primarily driven by the specificity of PSP in the ED and the sensitivity of PSP in the ICU.

**Conclusions:**

The results of the model indicate that PSP testing is cost saving compared to standard of care in diagnosis of sepsis. The abundance of sepsis cases in the ED and ICU make these findings important in the clinical field and further support the potential of sensitive and specific markers of sepsis to not only improve patient outcomes but also reduce healthcare expenditures.

## Introduction

According to the Third International Consensus Definitions for Sepsis and Septic Shock, sepsis is defined as life-threatening organ dysfunction caused by a dysregulated host response to infection [1]. A recent study estimated that there were 48.9 million sepsis cases across the globe in 2017, 11 million of which resulted in sepsis-related death, which is substantially greater than the number of worldwide deaths caused by tuberculosis (1.5 million deaths), Human Immunodeficiency Virus (HIV) (0.68 million deaths) and Malaria (0.63 million deaths) in 2020 [2]. The Centers for Disease Control and Prevention (CDC) estimate that 1.7 million adults in the United States develop sepsis each year [3, 4]. Sepsis is a common cause of morbidity and mortality in US hospitals, and it is a significant economic burden; it was the most expensive condition treated in US hospitals in 2013, costing the US nearly $24 billion [3, 5–7]. A 2020 analysis of Medicare claims found a 40% increase in sepsis-related inpatient hospital admissions between 2012 and 2018, and associated costs have risen from $17.8 billion to $22.4 billion [5].

Diagnosis of sepsis in the Emergency Department (ED) or Intensive Care Unit (ICU) can be challenging because the signs and symptoms are often nonspecific, and not all signs are present in all patients [1, 8]. Rapid treatment of patients with suspected infection decreases mortality but increases risk of antibiotic resistance and *clostridium difficile* infection (CDI), both of which are associated with longer hospital stays and higher costs [9, 10]. Indications of sepsis include fever, elevated heart rate, hyperventilation, high white blood cell counts and elevated inflammatory markers [8, 11]. The 2018 update to the International Guidelines for Management of Sepsis and Septic Shock recommended that physicians obtain a blood culture and lactate level measurement within one hour of admission if sepsis is suspected and immediately administer broad-spectrum antibiotics. These guidelines were designed based on evidence that early recognition and treatment of sepsis improves outcomes [12–15].

Although blood cultures and lactate serum levels are common diagnostic tools for identification of sepsis, they have significant drawbacks. Blood cultures have low sensitivity and are positive in only 30–40% of septic patients. Analysis of the culture can take 24 h or more, and recent antibiotic use may produce a false negative [8, 13, 16]. Elevated serum lactate levels are a sign of tissue hypoperfusion and are strongly associated with in-hospital sepsis mortality, but elevated lactate levels are non-specific to sepsis [13, 17, 18].

Sepsis host-response blood protein biomarkers may provide a more rapid and accurate means of identifying septic patients and monitoring treatment response [8, 19]. C-reactive protein (CRP) and procalcitonin (PCT) are two biomarkers used to diagnose sepsis in clinical practice. CRP is a sensitive but non-specific marker of infection, and may also rise in reaction to trauma or inflammatory disorders [8, 20, 21]. PCT has higher specificity than lactate or CRP, and can be used to assess severity of sepsis, but levels may also elevate in response to non-infectious causes, and not all studies support widespread use [8, 19–22]. Studies have shown that use of PCT testing is associated with a minor reduction in healthcare cost, shorter hospital stays, and reduced antibiotic use [23–25]. Sepsis-related mortality increases with every hour of delay prior to antibiotic administration, so rapid point-of-care diagnosis has the potential to reduce mortality in sepsis patients [26].

Pancreatic stone protein (PSP) is a novel biomarker that is more sensitive and specific to sepsis than CRP and PCT [21, 27, 28]. PSP levels may rise above the normal range in sepsis patients before other signs and symptoms appear, enabling earlier diagnosis and appropriate treatment of sepsis [27]. A rapid, accurate sepsis diagnostic has the potential to decrease mortality and costs associated with sepsis treatment. To date there are no economic studies on the implementation of rapid bedside PSP testing in ED or ICU settings in the United States. The objective of this study is to develop a cost-impact model for the use of rapid PSP testing in these care settings.

## Materials and methods

### Model design and parameters

**Fig. 1:**
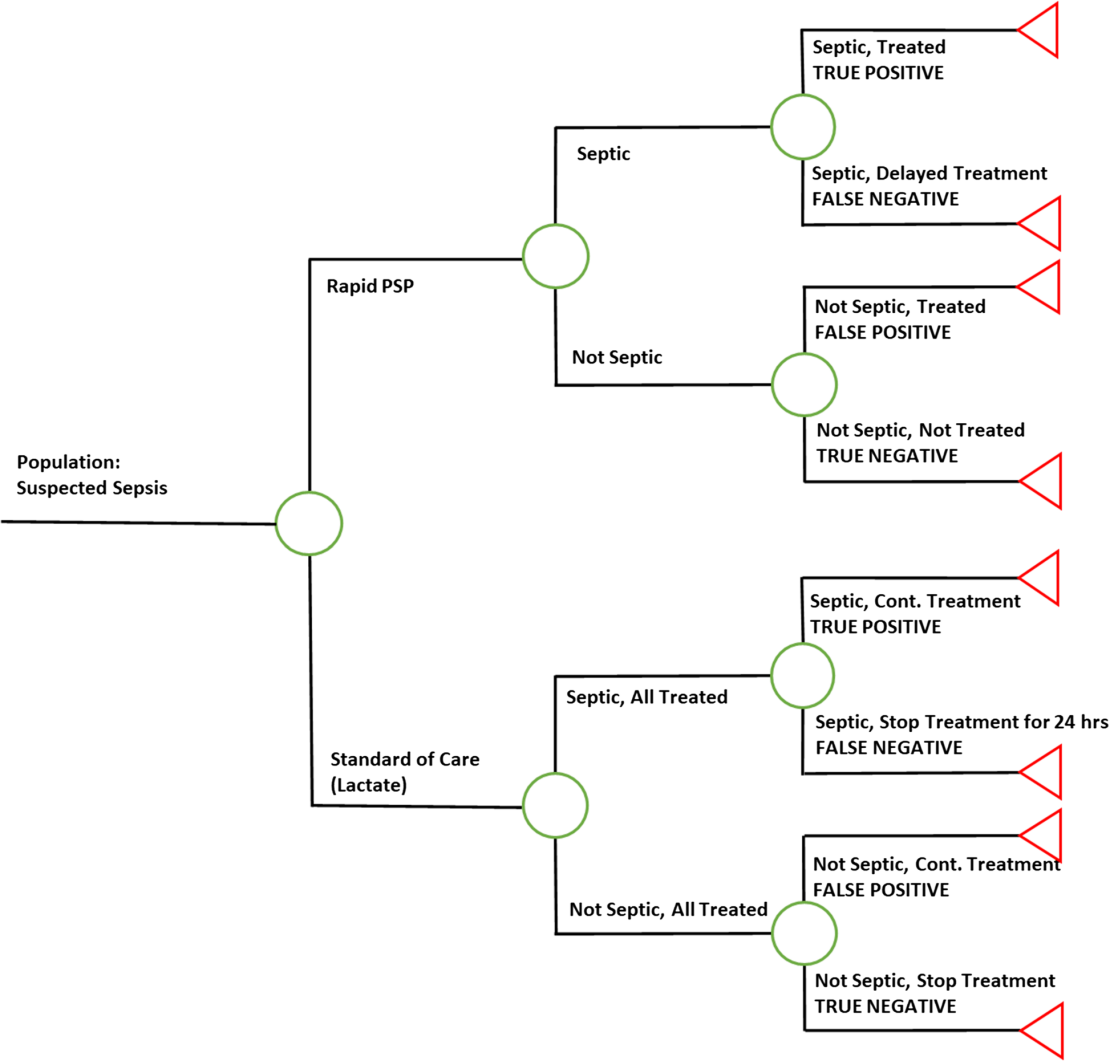
Decision tree model for diagnosis of sepsis. Abbreviations: PSP = pancreatic stone protein

The model is a decision tree based on the diagnostic and care pathway for a patient presenting to the ED or ICU with signs and symptoms of sepsis (Fig. 1). The model design and key parameters were developed based on a thorough review of sepsis research, including literature on sepsis modelling methodology, clinical outcomes, and economic outcomes. The initial search also included other types of infections typically associated with sepsis such as respiratory tract infections. The review focused on studies conducted from a US perspective or using US hospital data in the ICU and ED. The model inputs and parameters were extracted from large US studies wherever possible.

Literature on the economic impact of the PSP biomarker in the diagnosis of sepsis is scarce, so the model in this study was developed based primarily on the expected impact of sensitivity and specificity improvements in comparison to the standard of care diagnostic. As PCT is a biomarker with similar diagnostic properties which would likely target the same populations as PSP, the control arms from PCT economic evaluations were often utilized to populate the standard of care arm of the model.

In the model, patients are assigned to either “standard of care” diagnostic arm or the rapid PSP diagnostic arm. The standard of care diagnostic arm follows the Surviving Sepsis Campaign guidelines: blood culture collection, serum lactate test, and immediate administration of broad-spectrum antibiotics. The model assumes that broad-spectrum antibiotics will be administered to all patients in the standard of care arm during a 24-h wait for blood culture results. The antibiotics are discontinued on the second day of care if blood culture results are negative after another 24-h wait for results. The rapid PSP diagnostic arm assumes collection of a blood culture, administration of a serum lactate test, and administration of the rapid PSP test. As a rapid point-of care test, PSP allows physicians to quickly rule out non-septic patients and immediately initiate antibiotics for patients with positive test results. Therefore, a PSP test can lead to an estimated 48-h, or 2-day, earlier identification of ineffective antibiotics in comparison to standard of care.

This model was used to estimate healthcare costs in two separate care settings: the ED and the ICU. Biomarker studies conducted in both care settings showed key differences in the “true positive” incidence of sepsis among patients with suspected infection, 30-day readmission for infection, and the daily cost of a hospital stay in either the general ward or in the more expensive ICU.

### Diagnostic sensitivity and specificity

The economic benefit from PSP diagnostics is attributable to the improved diagnostic accuracy in comparison to standard of care. Four distinct test results are possible for each diagnostic arm of the model: true positive, false positive, true negative, and false negative. Septic patients who are diagnosed appropriately (i.e., true positive) undergo a full-length hospital stay and treatment with antibiotics [26, 28]. Non-septic patients diagnosed appropriately (i.e., true negative) under the current standard of care receive a single day of antibiotic treatment while awaiting results from blood cultures, after which antibiotics are discontinued, whereas those identified by rapid PSP testing are never treated with antibiotics. Skoglund and colleagues (2019) documented a difference of 4 fewer days in hospital length of stay (LOS) for patients with a negative blood culture compared to those with true bacteremia [29]. The model assumes that sepsis tests are repeated once every 24 h, so a false positive or false negative will be detected on the second day of treatment and delays the detection of appropriate test results by one day. False positive results lead to unnecessary initiation of antibiotic treatment and delay true negative results by one day, leading to a 2-day length of treatment and a difference of 3 fewer LOS days (4–1) compared to true positive patients. A false negative test result delays a true positive result by one day, increasing the LOS by one day. Patients still receive a full-length of treatment but the delay in proper antibiotic treatment increases the risk of severe sepsis and therefore the probability of readmission by an estimated 2.1% [7]. The impact of test results by sensitivity and specificity on length of hospital stay, antibiotic treatment length, and readmission rates are summarized in Table 1.

**Table 1:** Effect of test sensitivity and specificity on model inputs

Test Result	Description	Model Representation	Source
True Positive	Patient Septic	LOS: Full-length hospital stay	Mewes 2019 & Balk 2017 [23, 25]
		Treatment length: Full-length	Voermans 2019 & Balk 2017 [24, 25]
		Readmission: Average	Broyles 2017 [30]
True Negative	Patient Not Septic	LOS: Reduced by 4 days^a^	Skoglund et al. 2019 [29]
		Treatment Length: 1 day SOC; 0 days rapid PSP	Assumption (antibiotics initiated until lactate results are received under SOC; No antibiotics under PSP)
		Readmission: None	Assumption (patient not septic)
False Positive	Patient Not Septic	LOS: Reduced by 3 days^a^	Assumption (delays true negative by one day)
		Treatment Length: 2 days	Assumption (treated for one day in addition to the day of treatment prior to initial lactate results before detecting false positive with second test)
		Readmission: None	Assumption (patient not septic)
False Negative	Patient septic with increased risk of severe sepsis	LOS: Increased by 1 day^a^	Assumption (delays true positive by one day)
		Treatment Length: Full-length	Assumption (full treatment required)
		Readmission: Average + 2.1%	Paoli 2018 [7]

^a^compared to full-length hospital stay, described in *model parameters*

*LOS* Length of stay, *SOC* Standard of care, *PSP* Pancreatic stone protein

### Model parameters

Key model parameters were chosen based on the literature review, and included clinical and economic inputs. Clinical inputs were summarized in Table 2 and included hospital and ICU length of stay, antibiotic days of therapy (number of days patient was on antibiotics), incidence of sepsis, probability of 30-day readmission due to infection, sensitivity and specificity of sepsis testing, and probability of and additional length of stay due to antibiotic resistance and CDI. Economic inputs were summarized in Table 3 and included cost of sepsis testing, hospital stays, and hospital readmissions**.**

**Table 2:** Baseline clinical inputs

Parameter	Standard of Care	Rapid PSP	Source
**Base Parameters**			
Length of stay on the regular ward (days; TP)^a^	6.5		Average of Mewes 2019, Balk 2017 [23, 25]
Length of stay in the ICU (days; TP)^a^	10.6		Average of Voermans 2019, Mewes 2019, Balk 2017 [23–25]
Antibiotic days of therapy	19.0		Average of Voermans 2019, Balk 2017 [24, 25]
**Emergency Department**			
Incidence of sepsis among patients presenting with infection	41%		Rhee 2017 [3]
30-day readmission for infection	20%		Broyles 2017 [30]
Patients admitted to the ICU	8%		Broyles 2017 [30]
**Intensive Care Unit**			
Incidence of sepsis among patients	77%		Average of Vincent 2009, Johnson 2018 [31, 32]
30-day readmission for infection	28%		Bishop 2014 [33]
**Sensitivity & Specificity**			
Test sensitivity	34%	85%	PSP: Average of Llewelyn 2013, Garcia de Guadiana-Romualdo 2017 [21, 28]; SOC: Singer 2014 [34]
Test specificity	82%	80%	PSP: Average of Llewelyn 2013, Garcia de Guadiana-Romualdo 2017 [21, 28]; SOC: Singer 2014 [34]
**Antibiotic Resistance (ABR)**			
Prevalence of ABR	19.40%		Burnham 2015 [9]
Percent Reduction in ABR per percent reduced antibiotic days	3.20%		Mewes 2019 [23]
Additional length of stay due to ABR^a^	4.6		Mewes 2019; Voermans 2019 [23, 24]
***Clostridium difficile*** ** infection (CDI)**			
Prevalence of CDI	3%		Mewes 2019 [23]
Additional length of stay due to CDI	5.85		Average of Mewes 2019, Voermans 2019 [23, 24]

^a^Value represents the baseline input; additional calculations specific to diagnostic arm were applied as described in the “Methods” section

**Table 3:** Economic inputs

Parameter	Cost (2020 USD)	Source
Antibiotic therapy per day	$176.37	Voermans 2019 [24]
General ward per day	$1646.53	Voermans 2019 [24]
ICU per day	$2021.22	Mewes 2019 [26]
Blood culture	$56.16	Voermans 2019 [24]
Rapid PSP test	$52.17	Mewes 2019 [23]
Lactate test	$36.22	Ward 2016 [35]
Hospital readmission	$17,705.66	Gadre 2018 [36]

The length of hospital stays and antibiotic treatment varied considerably between studies. Therefore, the values were averaged from the control arm of three PCT studies with highest quality [23–25]. The estimated baseline hospital stay was 6.5 days in the ED and 10.6 days in the ICU, and the length of antibiotic treatment was 19 days.

Sensitivity and specificity of SOC were estimated from a 2014 study by Singer et al. on the implementation of lactate testing alongside clinical judgment in a US ED (34 and 82%, respectively) [34]. Sensitivity and specificity of rapid PSP tests were estimated from two studies on the use of PSP testing for sepsis diagnosis in an ED and ICU setting (85% and 80%, respectively) [21, 28]. The majority of baseline parameters were assumed to be equal for each test outcome regardless of the diagnostic arm. However, the length of treatment differed between standard of care and rapid PSP when a TN result was achieved because standard of care-diagnosed patients initiated immediate antibiotics and thus experienced more treatment days. The increase in treatment days impacted the probabilities of antibacterial resistance (ABR) and CDI as well. The potential for early identification of hospital-onset sepsis for admitted patients was also quantified, reflecting the value of daily in-hospital PSP testing. This enables identification of sepsis before patients become symptomatic, which would precede the distribution of a lactate test with SOC. It was assumed that the general ward and ICU length of stay varied between treatment arms to reflect this potential early identification of hospital-onset sepsis for admitted patients (an estimated 13% of the population). For bacterial inpatients correctly identified with PSP (85% of the hospital onset patients in this arm), we attributed the normal baseline LOS of 6.5 days [7]. However, for patients correctly identified with standard of care (34% of the hospital onset patients in this arm), patients were attributed a 31% greater LOS in the ward and 26% greater LOS in the ICU (the respective differentials between sepsis without dysfunction and severe sepsis) compared to the baseline LOS [7]. This impact is a result of expected increase in patient severity due to an estimated 2-day delay in recognition with standard of care compared to rapid PSP, which results in a true positive (TP) LOS of 6.8 days in the ED and 10.9 days in the ICU for standard of care.

Any exposure to antibiotics leads to increased probability of antibiotic resistance and CDI. The baseline probability of antibiotic resistance was estimated from a US-based 2015 study of multidrug resistance in septic patients, which found that among 510 patients with confirmed sepsis, 19.4% met criteria for multidrug resistance [9]. In a study assessing the impact of PCT on patient outcomes, Mewes et al. estimated a 3.2% reduction in antibiotic resistance for each 1% reduction in antibiotic treatment days. Utilizing the baseline number of antibiotic treatment days, we calculated ABR probabilities for each test outcome as a function of the aligning antibiotic treatment lengths. The baseline probability of CDI was estimated from Mewes et al., and a CDI risk ratio of 1.34 for every 10% increase in antibiotic days was incorporated to calculate the varying CDI probabilities for each test outcome [37]. Cases of antibiotic resistance and CDI impacted costs by extending hospital length of length of stay by 4.60 days and 5.85 days respectively [23]. For patients on the PSP arm, we decrease the resistance-attributable LOS by 2 days to simulate the impact of an estimated 2-day earlier identification of ineffective antibiotics in comparison to standard of care. This impact was only attributed to positively identified (85%) septic patients (41% of patients in the ED and 77% of patients in the ICU) on the PSP arm to account for this impact being dependent on follow-up test results, not the result obtained upon patient presentation.

Economic inputs were estimated from the literature and costs were inflated to 2020 USD (Table 3). The cost of each PSP test was assumed to be equivalent to the cost of a PCT test because of lack of cost data on PSP testing.

### Model extrapolation

The national cost of sepsis is difficult to estimate due to uncertainty around the incidence of sepsis in the United States, which varies depending on the criteria and data sources used to identify sepsis. Claims analyses may identify septic patients through explicit ICD-10 codes and may also include codes for organ dysfunction and infection that imply sepsis [3, 7]. Clinical data analyses may use different clinical criteria to identify sepsis including Sequential Organ Failure Assessment (SOFA) score, quick SOFA score, and Systemic Inflammatory Response Syndrome criteria [3, 38, 39].

An analysis of 2013 inpatient hospital data from the Healthcare Cost and Utilization Project found that sepsis cost the US $23.7 billion for 1.3 million hospital visits in 2013 [6]. To assess the potential impact of a rapid PSP diagnostic on the national level, we multiplied the average cost of care for truly septic patients by the estimated incidence of sepsis. This includes true positive and false negative patient diagnoses. Estimates were also calculated separately for ED and ICU settings.

Current estimates of the national cost of sepsis are specific to patients with confirmed sepsis, and do not consider the additional costs associated with monitoring for sepsis. Rhee et al. estimated that only 41% of those with presumed serious infection are truly septic [3]. Therefore, we assumed that the number of truly septic patients estimated by the CDC represents 41% of the total patients monitored. This incidence was multiplied by the expected cost of monitoring non-septic patients with suspected infection to calculate the annual cost of sepsis. This includes subjects in the true negative and false positive arms of the model.

### Sensitivity analysis

A sensitivity analysis was conducted by varying each parameter by 15% in order to determine the cost drivers of each model. No reliable or available uncertainty parameters could be identified from the literature. A 15% variation was assumed to likely account for any reasonable variation in inputs parameters. This analysis was also done separately for ED and ICU settings.

## Results

The expected per-person cost of standard of care-guided treatment was $14,515 in the ED and $42,464 in the ICU. Rapid PSP-guided treatment cost was $12,827 in the ED and $39,148 in the ICU. Use of rapid PSP testing instead of standard of care would save $1688 in the ED and $3315 in the ICU setting. A false negative test result was the most expensive diagnostic pathway for both model settings because of the consequences of delayed treatment. A true negative result was the least expensive diagnostic pathway because no treatment was required and patients were discharged quickly.

The national estimates are shown in Table 4. The average cost of care for truly septic patients was $22,177 for PSP testing and $24,023 for standard of care. Extrapolating costs using the CDC estimate of 1.7 million sepsis cases per year, the study finds that sepsis care guided by standard diagnostics costs the US $40.8 billion, while PSP-guided care costs $37.7 billion, representing an annual savings of $3.1 billion.

**Table 4:** National estimates

	Cost of Care per Patient (USD)	Estimated National Cost of Care (USD)^a^
Overall		
True Sepsis^b^		
Standard of Care	24,023	40.8 billion
PSP	22,177	37.7 billion
Potential Savings	1847	3.1 billion
Monitoring Non-Septic Patients^c^		
Standard of Care	7907	19.4 billion
PSP	6329	15.5 billion
Potential Savings	1578	3.9 billion
Total Potential Savings (National level)		7.0 billion
ED and General Ward		
True Sepsis^b^		
Standard of Care	22,149	37.7 billion
PSP	20,445	34.8 billion
Potential Savings	1704	2.9 billion
Monitoring Non-Septic Patients^c^		
Standard of Care	6781	16.6 billion
PSP	5234	12.8 billion
Potential Savings	1547	3.8 billion
Total Potential Savings (National level)		6.7 billion
ICU		
True Sepsis^b^		
Standard of Care	25,775	3.5 billion
PSP	24,243	3.3 billion
Potential Savings	1532	208.3 million
Monitoring Non-Septic Patients^c^		
Standard of Care	15,331	623 million
PSP	15,118	614 million
Potential Savings	213	8.7 million
Total Potential Savings (National level)		217.0 million

^a^1.7 million * cost of care per septic patient for ED model; 2.4 million * cost of monitoring per non-septic patient for ED model; 136,000 * cost of care per septic patient for ICU model; 40,623* cost of monitoring per non-septic patient for ICU model

^b^True positive, false negative

^c^False positive, true negative

We estimate 4.1 million people are monitored for sepsis and 2.4 million patients are not truly septic. Monitoring non-septic patients is estimated to cost the US $19.4 billion for standard diagnostics and $15.5 billion for rapid PSP testing. The annual savings associated with monitoring non-septic patients with rapid PSP testing rather than standard diagnosis is $3.9 billion. Total potential savings for monitoring sepsis with rapid PSP testing was 6.7 billion for ED patients and 217 million for ICU patients.

**Fig. 2:**
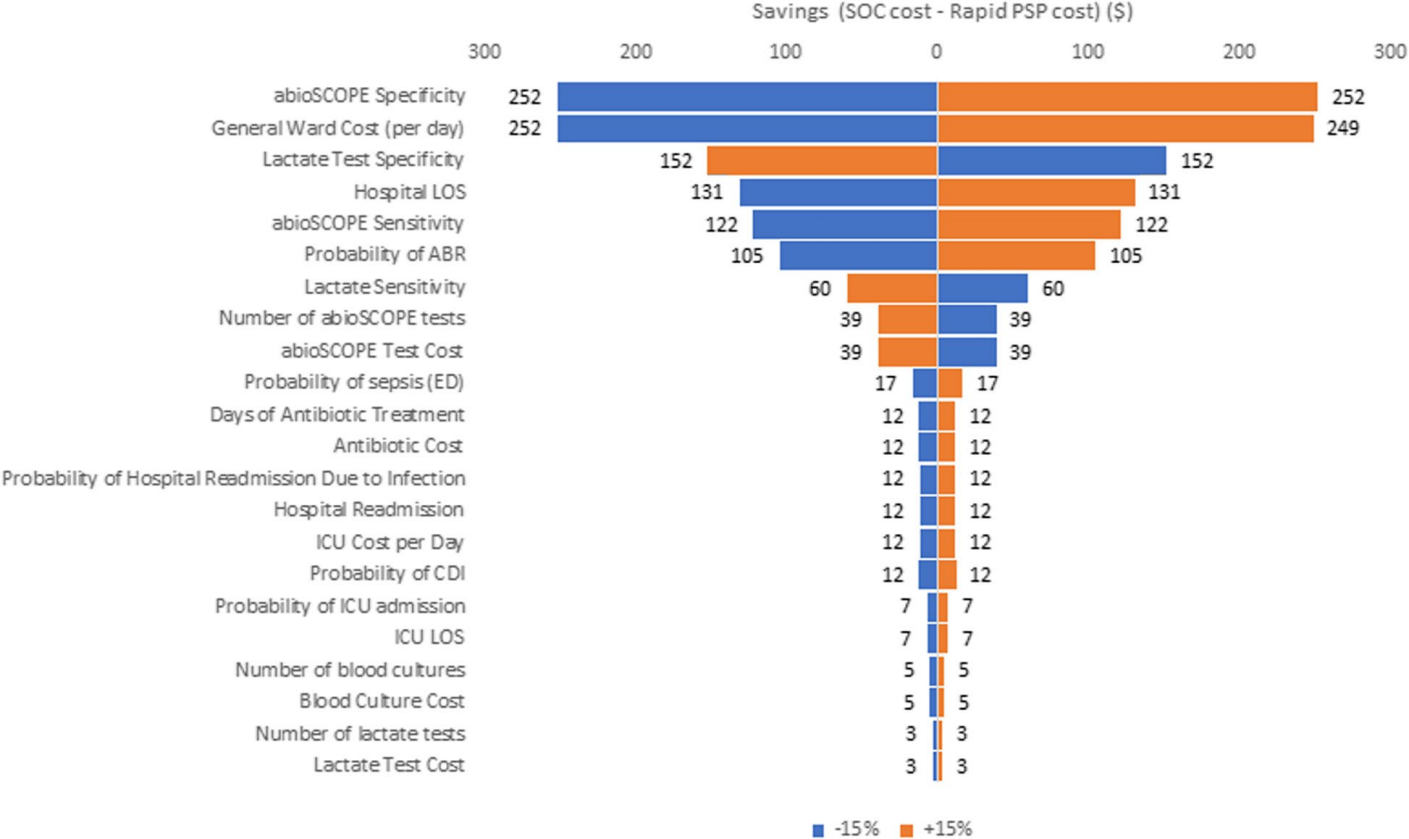
ED sensitivity analysis. Abbreviations: ICU = intensive care unit; PSP = pancreatic stone protein; SOC = standard of care

**Fig. 3:**
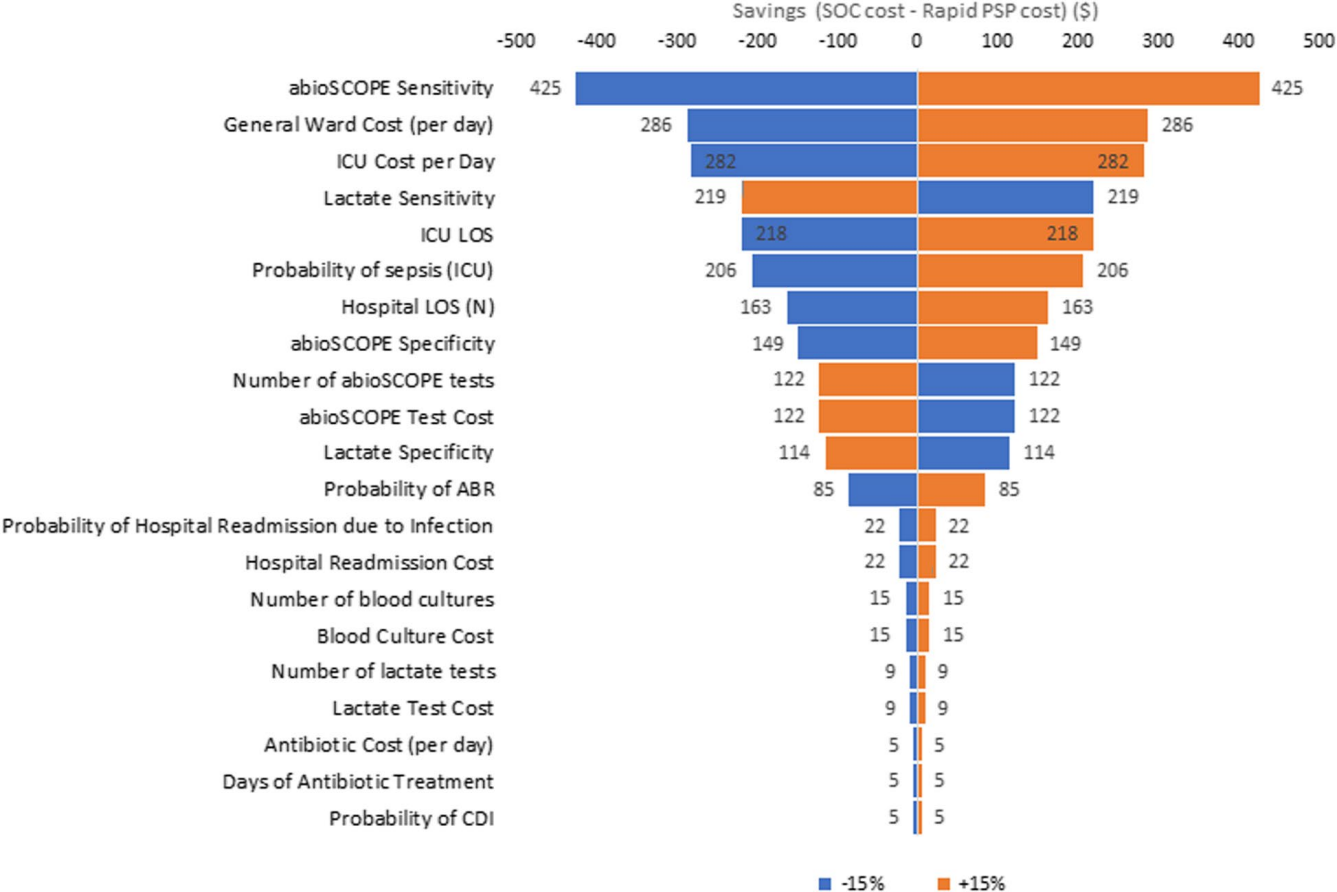
ICU sensitivity analysis. Abbreviations: CDI = Clostridioides difficile; ICU = intensive care unit; LOS = length of stay; PSP = pancreatic stone protein; SOC = standard of care

The sensitivity analysis was presented in Figs. 2 and 3. The ED model was most sensitive to changes in PSP test specificity, daily general ward cost, lactate test specificity, and hospital length of stay. 15% increases in each of these parameters influenced cost savings associated with PSP by $252, $249, -$152, and $131, respectively, compared to standard of care. In the ICU setting, the model was most sensitive to changes in PSP test sensitivity, daily general ward cost, the ICU cost per day, and lactate sensitivity. 15% increases in each of these parameters influenced savings associated with PSP by $425, $286, $282 and -$219 respectively.

## Discussion

Rapid PSP testing may be used to rapidly diagnose sepsis, stratify by disease severity and prognosis, and guide appropriate initiation or de-escalation of treatment [27]. These characteristics generate downstream benefits in terms of healthcare costs and patient outcomes. The results of this study are comparable to economic studies of PCT testing in the ICU. The estimated total cost of PCT-guided sepsis care ranged from $30,454 to $40,597 depending on the parameters considered and severity of sepsis [23, 25, 40]. This suggests that an estimated cost of $39,148 for PSP-guided sepsis treatment is reasonable. The costs associated with standard of care diagnostics are also comparable to other studies. The current study’s estimate of $42,464 is within the cost range of $18,000 to $51,000 per admission depending on severity and patient risk factors [7].

The sensitivity analysis showed that overall savings vary considerably with the average cost of a general ward stay and cost of an ICU stay. These are the highest itemized costs considered in the model and every patient incurs the cost. Sepsis-related ICU costs found in literature ranged from $1893 to $1981 per day, and general ward costs ranged from $1305 to $1500 [23–25, 29, 41].

The importance of test specificity in the ED model is unsurprising because test results determine the resulting care pathway, which affects major cost drivers like length of stay. Test sensitivity has a greater impact in the ICU model because true sepsis is more prevalent in the ICU, which leads to a larger emphasis on avoiding false negative diagnostics [3, 31, 32].

Sepsis is a medical emergency that requires prompt recognition and clinical management. The Surviving Sepsis Campaign developed one- and three-hour care bundles to guide rapid clinical management of suspected sepsis [13]. Availability of a rapid bedside sepsis test will reduce the time to sepsis identification and treatment, which will reduce downstream healthcare costs and mortality rates. A point-of-care PSP test (Abionic SA, Epalinges, Switzerland) has already received CE marking and is currently available in selected countries.

The incidence of sepsis is rising with time; the number of sepsis-related hospital stays nearly tripled between 2005 and 2014 [42]. The lowest estimated sepsis incidence in the United States was 570,000 cases per year, and the highest was 3.1 million cases per year [3]. When these incidences are multiplied by the estimated ED standard of care cost of $24,023 per patient with true sepsis, the national cost of sepsis is estimated from $14 billion to $75 billion, which is a considerable range.

Sepsis is a global problem with similar incidences in developed countries and even greater incidences in developing countries [2]. The World Health Organization (WHO) recognizes sepsis as a global health priority and adopted a resolution urging the 194 United Nations Member States to improve the prevention, diagnosis, and management of sepsis [43]. Furthermore, antimicrobial resistance (AMR) is a major problem in developing countries and was declared by the WHO as one of the top 10 global public health threats [43]. Although the model uses US parameters derived from the literature, we expect the implications of this study to be applicable in other healthcare systems worldwide. However, we do not expect a direct cost comparison of savings because this study used US parameters that may differ across countries. Future research would entail using parameter values from the international literature to compare findings and create country-specific model adaptations.

A limitation of this model is that indirect cost savings associated with rapid PSP testing are not considered. These potential savings include reduced work absenteeism, the societal cost of death, and improved ED efficiency. Absenteeism would likely correlate with hospital length of stay, which is a driver of cost savings in the PSP diagnostic arm. The rapidity and diagnostic accuracy of a point-of-care PSP test would improve ED efficiency, reduce crowding, and avoid costs associated with misdiagnosis, delay to treatment and decline in procedural accuracy [44–46]. It would also prevent many missed diagnoses due to improved sensitivity, thereby potentially limiting the number of severity-related deaths from delayed treatment.

## Conclusion

The goal of this study was to develop a cost-impact model for the use of rapid PSP testing in US ED and ICU settings. The results of the model indicate that use of point-of-care PSP testing is cost saving compared to standard of care in diagnosis of sepsis. The rapid PSP test was found to reduce costs by $1688 per patient in the ED and $3315 per patient in the ICU compared to standard of care. This cost reduction was primarily driven by improved test sensitivity and specificity for PSP compared to standard of care. These results were extrapolated to the national population of septic patients. The estimated cost of sepsis was found to be US $40.8 billion for standard of care and $37.7 billion for rapid PSP-guided sepsis care, resulting in total cost savings of $3.1 billion.

## Data Availability

Data sharing is not applicable to this article as no datasets were generated or analysed during the current study.

## Abbreviations

CDC: Center for Disease Control and Prevention
ED: Emergency department
ICU: Intensive care unit
CDI: Clostiridium difficile infection
CRP: C-reactive protein
PCT: Procalcitonin
PSP: Pancreatic stone protein
TN: True negative
ABR: Antibacterial resistance
LOS: Length of stay
TP: True positive
SOFA: Sequential Organ Failure Assessment
